# Synergistic effects of neck circumference and metabolic risk factors on insulin resistance: the Cardiometabolic Risk in Chinese (CRC) study

**DOI:** 10.1186/1758-5996-6-116

**Published:** 2014-11-01

**Authors:** Jun Liang, Fei Teng, Xuekui Liu, Caiyan Zou, Yu Wang, Lianjun Dou, Zilin Sun, Lu Qi

**Affiliations:** Department of Endocrinology, Xuzhou Central Hospital, Xuzhou Clinical School of Xuzhou Medical College; Affiliated Hospital of Southeast University, 199# South Jiefang Road, Xuzhou, 221009 Jiangsu China; Institute of diabetes, Medical School, Southeast University, Nanjing, 210009 Jiangsu China; Department of Nutrition, Harvard School of Public Health, Boston, Massachusetts 02115 USA; Department of Medicine, Channing Laboratory, Brigham and Women’s Hospital and Harvard Medical School, Boston, Massachusetts 02115 USA

**Keywords:** Insulin resistance, Neck circumference, Uric acid, Triglyceride, Synergistic effects

## Abstract

**Objectives:**

Recent studies have associated neck circumference (NC) with insulin resistance (IR). We examined whether such relation was modified by other metabolic risk factors.

**Methods:**

The study samples were from a community-based health examination survey in central China. A total of 2588 apparently healthy Chinese men and women were included.

**Results:**

Plasma levels of total cholesterol (TC), HDL-C, uric acid (UA) and diastolic blood pressure (DBP) were independently associated with NC after adjusted for age, sex, body mass index (BMI), waist circumference (WC) and hip circumference (HC) (P = 0.009, 0.001, 0.015 and 0.015, respectively). We observed significant interactions of NC with triglyceride (TG) and UA (all the p for interaction = 0.001) in relation to HOMA-IR. It appeared that the associations between NC and HOMA-IR were more evident in those with higher UA or TG level.

**Conclusions:**

Our data indicate that in apparently healthy Chinese adults, there were synergistic effects of UA, TG and neck circumference on insulin resistance.

## Background

Neck circumference is a proxy for upper-body fat and a reliable, simple, time saving screening measure, among numerous others such as BMI and WC, for identification of individuals with excess body fat or its abnormal distribution. In Framingham study, NC was positively associated with insulin resistance [1]. In our recent analysis, we found NC is independently related to IR in Chinese [2].

NC has been also related to various metabolic risk factors such as blood pressure and lipids [3, 4], independent of overall adiposity (body mass index) and central obesity (waist circumference or visceral adipose tissue) [1]. It remains unclear whether these risk factors modify the relation between NC and IR [5].

In the present study, we comprehensively analyzed the associations of NC and other metabolic risk factors, and their interactions in relation to insulin resistance in a large cohort of apparently healthy Chinese adults.

## Materials and methods

### Study population

In the Cardiometabolic Risk in Chinese (CRC) Study, we performed a community-based health examination survey for 6,431 individuals (18–93 y) who were randomly selected from residents living in the urban area of central China, in 2009. The details of this study have been presented elsewhere [6–9]. Written consents were obtained from all the participants. The CRC study was reviewed and approved by the ethics committee of the Central Hospital of Xuzhou, China and sponsored by Jiangsu Health International Exchange Program. NC was not measured in all the CRC participants, for the present study, we included adult men and women (≥20 y) who were successfully measured NC, and relevant cardiometabolic markers including fasting glucose, insulin, lipids, blood pressure, BMI, WC. We excluded the potential patients with history of diabetes and those with fasting glucose ≥ 7.0 mmol/L and/or 2 h OGTT ≥11.1 mmol/L and/or HbA1c ≥ 6.5%, also excluded subjects with goiter and other neck masses and deformity with ultrasound. In total 2588 subjects were included. There was no significant difference in the clinical characteristics between the participants of the present analysis and those who were not included.

### Anthropometric measures

NC (cm) was measured with head erect and eyes facing forward, horizontally at the upper margin of the laryngeal prominence with a flexible tape [2]. WC (cm) was measured to the nearest 1 cm in the horizontal plane at the midpoint between the lowest rib and the iliac crest. HC (cm) was measured at the level of maximal protrusion of the gluteal muscles. ALL the human body dimensions have three readings and taken the average. WHR was calculated as WC divided by HC. Height and body weight were measured with participants standing without shoes and heavy outer garments. BMI was calculated as weight (in kilograms) divided by height (in meters) squared. Percentage of body fat was estimated with bioelectrical impedance, using the Omron Body Fat Analyzer HBF-306. Subjects with a body fat percentage measured by bipolar bioelectrical impedance analysis (BF% (IMP)) < or = 20.9% were considered normal weight, while subjects with a BF% (IMP) > or = 21.0% were considered overweight. Blood pressure (BP) was measured after the subject had rested for at least 5 minutes with a mercury manometer by doctors. Three measurements, 60 seconds apart, were taken. The mean of the three measurements was used for analysis.

### Assessment of biomarkers

Venous blood sample was drawn from all subjects after an overnight fast (8–12 h). After blood was drawn, specimens were allowed to clot at room temperature for 1-3 h and serum was separated. Immediately following clotting serum was separated by centrifugation for 15 min at 3,000 rpm. Fasting blood specimens were collected for measurement of uric acid (UA), glucose, total cholesterol (TC), triglyceride (TG), high density lipoprotein cholesterol (HDL-C), and low density lipoprotein cholesterol (LDL-C). All biochemical assays were determined by enzymatically on an autoanalyzer (Type 7600, Hitachi Ltd, Tokyo, Japan). Serum insulin concentration was determined by competitive radioimmunoassay (Roche, E170, Germany).

Insulin Resistance was assessed using the Homeostasis Model Assessment-Insulin Resistance (HOMA-IR) index, derived from plasma glucose and insulin [10]. According to recommendations from the European Group for the Study of Insulin Resistance, the HOMA-IR index was calculated according to the formula: Fasting plasma glucose (mmol/l) × Fasting serum insulin (U/ml) /22.5. By convention, individuals in the upper quartile in HOMA –IR index for non-diabetics are defined as insulin resistance [11].

### Statistical analyses

Fasting glucose, insulin, TG and TC levels were logarithmically transformed to improve the normality. Linear regression models were used to evaluate associations between NC (in sex-specific quintiles) and metabolic markers, adjusting for covariates. Interactions between NC and metabolic risk factors were tested by introduction of the cross-product terms in the linear regression models. All reported P values were two tailed. Variables with P values of <0.05 were considered statistically significant. Data management and statistical analysis were conducted using SAS statistical software (SAS Institute Inc., Cary, NC, USA).

## Results

### The characteristics of the study participants by neck circumference

In total 2588 apparently healthy Chinese adults were included in the present study. The mean age of study population was 44.3 (20–87) years and the mean BMI was 24.4 kg/m^2^. Table 1 shows the characteristics of the study participants according to NC levels (in quintiles). NC was positively related to BMI, WC, HC, and WHR (all p < 0.0001).

**Table 1:** **Characteristics of participants by NC in quintiles**

Variables	NC (in quintiles, cm)					
	Q1(≤33)	Q2(34–35)	Q3(36–37)	Q4(38–39)	Q5(≥40)	P for trend
N	801	455	427	517	388	
Age, years	43.1 ± 11.3	45.3 ± 12.1	43.3 ± 12.9	44.5 ± 11.3	43.4 ± 9.5	<0.0001
BMI, kg/m^2^	21.9 ± 2.4	23.1 ± 2.9	24.3 ± 2.1	25.8 ± 2.1	27.8 ± 2.6	<0.0001
BF, %	28.8 ± 4.8	26.7 ± 6.5	25.1 ± 4.6	26.1 ± 4.2	27.4 ± 3.9	<0.0001
WC, cm	75.5 ± 6.7	81.5 ± 6.3	86.4 ± 5.5	91.3 ± 5.9	97.0 ± 6.9	<0.0001
HC, cm	92.5 ± 5.1	95.0 ± 5.2	96.9 ± 4.3	99.9 ± 4.6	104.4 ± 5.2	<0.0001
WHR, %	0.82 ± 0.06	0.86 ± 0.05	0.89 ± 0.04	0.91 ± 0.05	0.93 ± 0.05	<0.0001

The values are presented as mean ± standard deviation.

*Abbreviations*: *BMI* Body mass index, *BF* Percent, body fat rate, *WC* Waist circumference, *HC* Hip circumference, *WHR* Waist/hip ratio.

### Association of neck circumference with metabolic risk factors

After adjustment for age and sex (Table 2), TC, TG, HDL-C, LDL-C, UA, SBP and DBP were independently associated with NC (all the P = 0.0001). In the models further adjusting for BMI, WC and HC, the associations of NC with TG, HDL-C, UA and DBP remained significant (P = 0.009, 0.001, 0.015 and 0.015, respectively). The difference in these markers between the two extreme groups quintiles 1(Q1) and quintiles 5(Q5) were 1.26 mmol/L, 0.34 mmol/L, 118.56, μmol/L and 12 mmHg respectively for TG, HDL-C, UA and DBP.

**Table 2:** **Associations between NC and biochemical risk factors**

	NC (in quintiles, cm)						
Variables	Q1(≤33)	Q2(34–35)	Q3(36–37)	Q4(38–39)	Q5(≥40)	P1	P2
TC, mmol/L	4.90 ± 0.86	4.92 ± 0.86	5.04 ± 0.87	5.10 ± 0.86	5.18 ± 0.93	0.0001	0.646
TG, mmol/L	1.03 ± 0.95	1.32 ± 1.06	1.71 ± 1.61	2.05 ± 1.68	2.29 ± 1.99	0.0001	0.009
HDL-C, mmol/L	1.44 ± 0.29	1.31 ± 0.29	1.22 ± 0.27	1.13 ± 0.56	1.10 ± 0.24	0.0001	0.001
LDL-C, mmol/L	2.84 ± 0.69	2.91 ± 0.72	3.02 ± 0.78	3.05 ± 0.75	3.11 ± 0.79	0.0001	0.909
UA, μmol/L	233.97 ± 53.55	277.68 ± 67.89	319.40 ± 65.53	338.75 ± 71.79	352.53 ± 75.39	0.0001	0.015
SBP, mmHg	115 ± 14	121 ± 15	125 ± 14	127 ± 16	131 ± 15	0.0001	0.843
DBP, mmHg	73 ± 10	77 ± 7	80 ± 10	82 ± 11	85 ± 11	0.0001	0.015

The values are presented as mean ± standard deviation.

*Abbreviations*: *TC* Total cholesterol, *TG* Triglyceride, *HDL-C* High density lipoprotein cholesterol, *LDL-C* Low density lipoprotein cholesterol, *UA* Uric acid, *SBP* Systolic blood pressure, *DBP* Diastolic blood pressure.

P1 was for models with age and sex adjusted; P2 was for models with age, sex, BMI, WC, and HC adjusted.

### Synergistic effects of serum uric acid, TG and NC on insulin resistance

**Figure 1:**
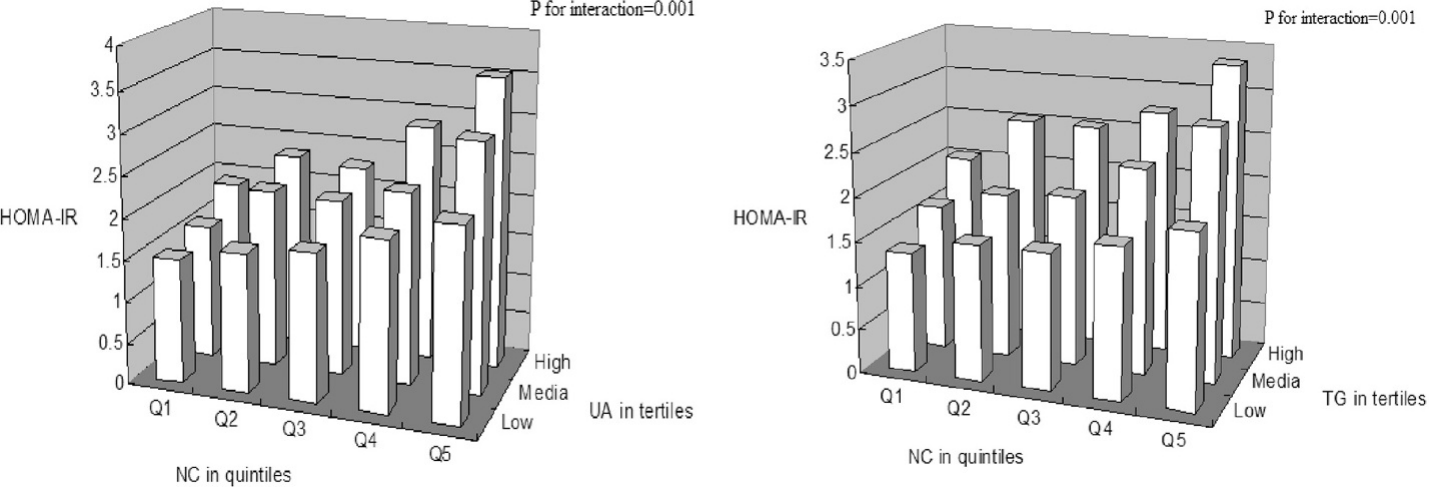
**Synergistic effects of SUA, TG and NC on insulin resistance.**

We further tested the interactions between NC (in quintiles) and these metabolic risk factors, and found significant interactions for TG and UA (both in tertiles) in relation to HOMA-IR (all the P for interaction = 0.001). Figure 1 shows the joint effects of NC and UA, as well as NC and TG in relation to HOMA-IR. For both UA and TG, we found that the associations between NC and HOMA-IR were more evident among those with higher levels of these markers.

## Discussion

In a large cohort of apparently healthy Chinese adults, we found that NC was related to metabolic risk factors including TG, HDL-C, UA and DBP. In our previous study, we found significant association between NC and increasing trend of HOMA-IR, even after adjusting for age and other cardiometabolic risk factors [2]. In this study, we found that plasma levels of UA or TG modified the relation between NC and HOMA-IR.

Our findings are in line with several recent studies in Caucasians and Chinese diabetic patients. Selim et al. found that NC was associated with HDL, FBG, TC, TG and blood pressure in Turkey obese children [4]. In the Framingham study, it was found that NC was associated with insulin resistance and cardiovascular risk factors. After adjustment for visceral adipose tissue, NC was positively associated with SBP, DBP, TC and FBG [1]. Locally acting fat depots may contribute to obesity complications and cardiovascular diseases, through direct paracrine effects, and the cardiovascular risk conferred especially by visceral and upper body adiposity. The carotid arteries are encased in fat, and total upper-body subcutaneous fat is estimated by NC. The recent study highlight NC has been independently correlated with cardiometabolic risk factors above and beyond that of other adiposity measures [12, 13].

Several mechanisms may be underlying the associations of high NC and metabolic risk factors. It has been documented that high NC was a significant predictor of obstructive sleep apnea syndrome (OSAS) [3], which has been associated with aggravates glycemic control, even at the earliest stages of glucose intolerance. In addition, intermittent hypoxemia and sleep fragmentation increases the risk of IR [14]. Our data, together with evidence from other studies, suggest that body fat accumulated in upper body segment may also contribute to the adverse consequence.

The synergistic effects of NC with UA and TG on IR remained unclear. Hyperuricemia has been associated with insulin resistance [15], and excess adiposity [16]. In our previous analysis, we found high UA levels were related to various metabolic disorders [8]. High-sensitivity C-reactive protein (hs-CRP) level is often higher in hyperuricemic patients than in the general population, hs-CRP level was found to be an independent predictor of homeostatic model assessment with insulin resistance [17]. In addition, soluble uric acid could increase tissue levels of NADPH oxidase and the generation of reactive oxygen species (ROS) in mature adipose tissue; oxidative-stressed adipose tissue shows decreased sensitivity to insulin as a risk factor of insulin resistance [18]. Uric acid levels have also been found to be positively correlated with the number of obstructive respiratory episodes and oxygen desaturations during sleep [19]. NC has been positively related to blood levels of TG [20]. It was recently found that NC was associated with atherogenic dyslipidaemia beyond BMI and waist circumference in both men and women [21]. Our data were consistent with an additive effect of NC and high levels of UA or TG on insulin resistance.

To our knowledge, study about the interactions of NC and metabolic risk factors on insulin resistance in general population of Chinese adults are lacking. The cross-sectional nature of the study to some extent limits its interpretation as to causality, prospective studies and follow-up data on our participants are warranted to confirm the causal relation. In addition, neck circumference is used to represent upper body subcutaneous fat, we did not perform radiographic measures to quantify this depot of fat directly. Physical fitness is very important on metabolic risk reduction, but in present study, we did not well analyze the interactions between metabolic risk factors and life style intervention. Finally, the study was performed in a Chinese population, further studies in other populations of different ethnicities are warranted to verify our findings.

In conclusion, we found significant associations of high NC with a variety of cardiometabolic risk factors including UA, TG, HDL-C, DBP in apparently healthy Chinese adults. In addition, we found significant synergistic effects of neck circumference with UA and TG on insulin resistance.

## Abbreviations

NC: Neck circumference
IR: Insulin resistance
HOMA-IR: Homeostasis Model Assessment-Insulin Resistance
BMI: Body mass index
BF%: Body fat rate
WC: Waist circumference
HC: Hip circumference
WHR: Waist/hip ratio
TC: Total cholesterol
TG: Triglyceride
HDL-C: High density lipoprotein cholesterol
LDL-C: Low density lipoprotein cholesterol
UA: Uric acid
SBP: Systolic blood pressure
DBP: Diastolic blood pressure.
